# Swallowing Improvement Surgery for Dysphagia in Wallenberg Syndrome: A Scoping Review of the English and Japanese Literature

**DOI:** 10.3390/jcm15187217

**Published:** 2026-09-17

**Authors:** Yi-An Lu, Rumi Ueha, Midori Nanamatsu, Cathrine Miura, Ryota Ishizuka, Naoyuki Matsumoto, Takao Goto, Kenji Kondo

**Affiliations:** 1 Department of Otolaryngology and Head and Neck Surgery, The University of Tokyo, Tokyo 113-8655, Japan; season49900@gmail.com (Y.-A.L.); nanamatsu.midori@gmail.com (M.N.); r_ishizuka_12@yahoo.co.jp (R.I.); smilenaoyuki24@gmail.com (N.M.); gottytakao@gmail.com (T.G.); kondok-tky@umin.ac.jp (K.K.); 2 Department of Otolaryngology Head and Neck Surgery, Linkou Chang Gung Memorial Hospital, Taoyuan 33305, Taiwan; 3 Swallowing Center, The University of Tokyo Hospital, Tokyo 113-8655, Japan; 4 Department of Otorhinolaryngology–Head and Neck Surgery, University of the East Ramon Magsaysay Memorial Medical Center, Quezon City 1113, Philippines; miura.cathrine@gmail.com

**Keywords:** Wallenberg syndrome, dysphagia, swallowing improvement surgery, cricopharyngeal myotomy, scoping review

## Abstract

**Background:** Dysphagia associated with Wallenberg syndrome (WS) often improves with swallowing rehabilitation; however, persistent cases may require surgical intervention. Various swallowing improvement procedures have been reported, but their strategies and outcomes remain unclear. This scoping review summarized the evidence on swallowing improvement surgery for WS. **Methods:** PubMed, Scopus, Web of Science, and Ichushi-Web were searched in accordance with the PRISMA-ScR framework. English- and Japanese-language studies reporting swallowing improvement surgery for WS were included. Patient characteristics, surgical procedures, outcomes, and complications were descriptively synthesized. **Results:** Twenty-one studies involving 53 patients met the inclusion criteria. Most patients had profound preoperative dysphagia requiring enteral feeding. Cricopharyngeal myotomy was performed in all patients, and at least 66.0% underwent combined procedures, most commonly with laryngeal suspension. Available postoperative data indicated improved oral intake in many patients, although outcome reporting was incomplete. Some patients required staged procedures because a single procedure did not adequately address their complex swallowing impairment. **Conclusions:** Reported outcomes suggest that swallowing improvement surgery may contribute to improved oral intake in selected patients with WS. The frequent use of combined procedures highlights the importance of individualized, pathophysiology-based surgical management. However, the evidence cannot distinguish surgical effects from spontaneous recovery, rehabilitation, or patient selection.

## 1. Introduction

Wallenberg syndrome (WS), also known as lateral medullary syndrome, is a brainstem stroke syndrome most commonly caused by ischemic infarction of the lateral medulla [1]. Vertebral artery dissection, hemorrhage, and other lesions involving the lateral medulla, however, may produce a similar clinical presentation with WS [1]. Patients typically present with a constellation of neurological deficits, including vertigo, ipsilateral limb ataxia, Horner syndrome, sensory disturbances, dysarthria, and dysphagia [1,2]. Among these manifestations, dysphagia is both common and clinically important. Although swallowing function often improves with neurological recovery and rehabilitation, dysphagia may persist in a subset of patients and become a major source of long-term disability [3,4]. Persistent swallowing impairment can lead to malnutrition, dehydration, aspiration pneumonia, and reduced quality of life. In severe cases, long-term enteral feeding and tracheostomy management may be required [5,6].

Swallowing impairments in WS are primarily attributed to disruption of medullary structures involved in swallowing control, including the nucleus ambiguus, nucleus tractus solitarius, and their associated neural pathways [3,7]. Consequently, patients may exhibit a combination of velopharyngeal insufficiency, weak pharyngeal contraction, reduced laryngeal elevation, delayed initiation of the swallowing reflex, laryngopharyngeal sensory deficit, and impaired upper esophageal sphincter (UES) opening [1,7]. These impairments often coexist and may contribute to pharyngeal residue, aspiration, and severe restrictions in oral intake [3,4]. The combination and severity of swallowing abnormalities vary considerably among patients, resulting in heterogeneous clinical presentations and management challenges.

While many patients improve with neurological recovery and rehabilitation, some continue to suffer from severe and persistent dysphagia. These patients may remain dependent on enteral feeding and are at increased risk of aspiration pneumonia. For selected patients with refractory dysphagia, swallowing improvement surgery may be considered to restore oral intake and improve quality of life [8,9]. Swallowing improvement surgeries comprise a variety of procedures targeting specific physiological impairments, including cricopharyngeal myotomy, laryngeal suspension, vocal fold medialization procedures, and pharyngeal reconstructive surgeries [10]. Despite growing recognition of swallowing improvement surgery as a treatment option for refractory dysphagia, its role in managing dysphagia associated with WS is not yet fully established. Consequently, evidence to support clinical decision-making and referral for surgical evaluation remains limited. Data regarding patient selection, timing of intervention, surgical procedures, and clinical outcomes is scattered across individual case reports and small case series. Notably, although surgical treatment for dysphagia associated with WS has been reported for more than three decades, much of the early literature originated from Japan and was published in Japanese-language journals [11,12,13]. Hence, these experiences have not been widely disseminated to the international medical community, and the literature remains fragmented and difficult to access.

Therefore, the aim of this scoping review was to systematically map the existing English- and Japanese-language literature on swallowing improvement surgery for dysphagia in WS. Specifically, we sought to summarize the characteristics of reported patients, timing of intervention, surgical procedures, postoperative swallowing outcomes, and complications reported in the literature.

## 2. Materials and Methods

This scoping review followed the Preferred Reporting Items for Systematic Reviews and Meta-Analyses extension for Scoping Reviews (PRISMA-ScR), 2018 [14]. The study protocol was not registered. The review question was developed according to the Joanna Briggs Institute (JBI) Evidence Synthesis framework, considering the population (patients with WS), concept (swallowing improvement surgery), and context (management of dysphagia) [15].

A search of PubMed, Scopus, Web of Science, and Ichushi-Web (Japan Medical Abstracts Society database) was conducted from database inception to August 2026. Search strategies combined terms related to WS, dysphagia, and swallowing improvement surgery using both free-text keywords and Boolean operators. The search strategy was as follows:

*PubMed, Scopus, Web of Science, and Ichushi-Web*

((“Wallenberg syndrome”) OR (“lateral medullary syndrome”) OR (“lateral medullary infarction”) OR (“lateral medullary stroke”)) AND ((dysphagia) OR (swallowing) OR (deglutition) OR (“swallowing disorder*”) OR (“deglutition disorder*”)) AND ((surgery) OR (surgical) OR (operation*) OR (“cricopharyngeal myotomy”) OR (“laryngeal suspension”) OR (“arytenoid adduction”) OR (thyroplasty) OR (“vocal fold medialization”) OR (“injection laryngoplasty”) OR (“pharyngeal flap”) OR (pharyngoplasty)).

*Reference-list screening*

In addition to the electronic database search, the reference lists of all included studies and relevant review articles were manually screened to identify additional eligible studies.

The predetermined inclusion criteria were: (1) patients diagnosed with WS or lateral medullary syndrome; (2) dysphagia requiring surgical intervention; (3) swallowing improvement surgery performed with the intention of improving swallowing function while preserving laryngeal function; (4) primary studies including case reports, case series, retrospective studies, and prospective observational studies; and (5) full-text articles published in English or Japanese.

Exclusion criteria were: (1) aspiration-prevention surgery, including total laryngectomy, laryngotracheal separation, tracheoesophageal diversion, and laryngeal closure procedures; (2) botulinum toxin injection into the cricopharyngeal muscle or endoscopic balloon dilation; (3) studies unrelated to dysphagia surgery; (4) review articles, editorials, letters, and expert opinions; (5) conference abstracts; (6) animal studies; and (7) articles written in languages other than English or Japanese. For the purpose of this review, botulinum toxin injection into the cricopharyngeal muscle and endoscopic balloon dilation were considered minimally invasive UES-directed interventions rather than swallowing improvement surgery because they do not involve surgical alteration of the swallowing-related anatomy. No chronological limitation was applied because of the rarity of surgical treatment for dysphagia associated with WS.

After removal of duplicate records, titles and abstracts were screened for eligibility. Full-text articles were subsequently reviewed according to the predefined inclusion and exclusion criteria. The study selection process is presented using a PRISMA flow diagram.

Data were extracted using a standardized data-charting form developed by the authors (YL, RU, and MN). Extracted variables included study characteristics (author, publication year, language, and study design); patient characteristics (number of patients, age, sex, lesion side, and etiology); dysphagia characteristics (time from onset to surgery, preoperative oral intake status, and tracheostomy status); surgical procedures (type of surgery and combined procedures); postoperative oral intake outcomes (pre- and post-operative Functional Oral Intake Scale [FOIS] [16] score when available or convertible from the original report); airway outcomes (tracheostomy status and stoma closure); and postoperative complications. When individual patient data were available, patient-level analyses were performed. For studies reporting only aggregate summary data, available information was extracted and summarized descriptively. Aggregate data were not incorporated into calculations of median values or interquartile ranges but were considered when describing overall study and patient characteristics.

Critical appraisal of included studies was not performed because the purpose of this scoping review was to map and summarize the existing literature rather than to assess methodological quality. Data were synthesized descriptively using tables and narrative summaries.

## 3. Results

**Figure 1:**
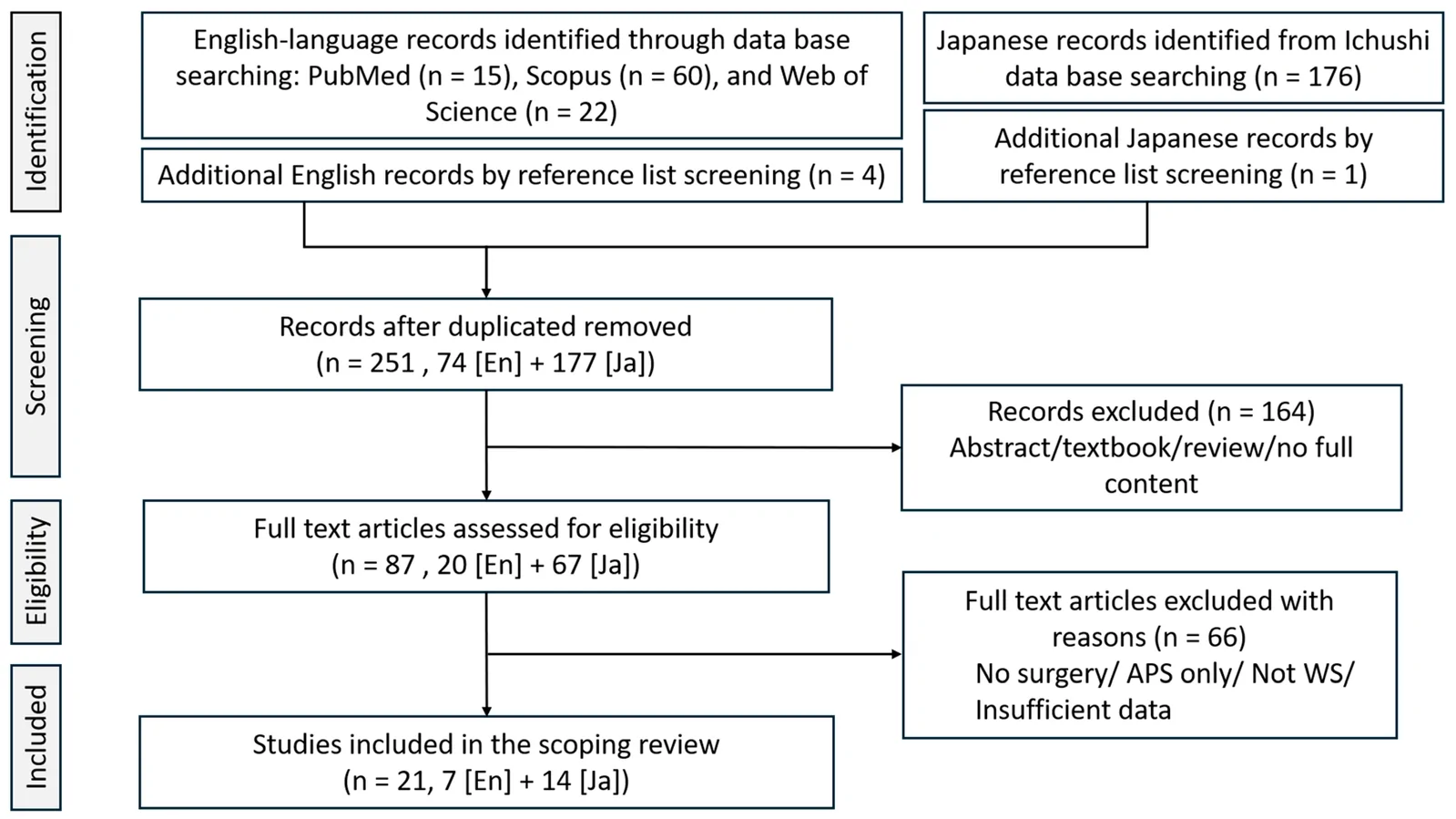
PRISMA flow diagram of study selection. En, English-language studies; Ja, Japanese-language studies; APS, aspiration-prevention surgery; WS, Wallenberg syndrome.

The literature search identified 273 records through database searching: 15 from PubMed, 60 from Scopus, 22 from Web of Science, and 176 from Ichushi-Web. An additional five records—four English-language and one Japanese-language record—were identified through reference-list screening. After duplicate removal, 251 records (74 English and 177 Japanese) remained for title and abstract screening. Of these, 164 were excluded, and 87 full-text articles (20 English and 67 Japanese) were assessed for eligibility. Following the exclusion of 66 full-text articles, 21 studies involving 53 eligible patients were included in the scoping review, comprising seven English-language and 14 Japanese-language studies. The study selection process is summarized in Figure 1.

### 3.1. Characteristics of Included Studies

The included studies were published between 1988 and 2024 (Table 1). Of the 21 included studies, 17 (81.0%) originated from Japan [9,10,11,12,13,17,18,19,20,21,22,23,24,25,26,27,28], 2 (9.5%) from India [29,30], 1 (4.8%) from the United States [31], and 1 (4.8%) from France [8]. Fourteen studies (66.7%) were published in Japanese and seven (33.3%) in English. Across the 21 studies, 53 eligible patients were included, with study-specific eligible sample sizes ranging from 1 to 19 patients. Twenty studies were case reports or case series, and one was a retrospective cohort study [9]. No randomized controlled trials, prospective interventional studies, or comparative studies were identified, highlighting the limited level of evidence currently available.

### 3.2. Patient Characteristics and Preoperative Clinical Data

A total of 53 patients with dysphagia secondary to WS were identified across the included studies (Table 2). Sex was reported for 50 patients (94.3%), including 46 males (92.0%) and 4 females (8.0%). Patient-level age data were available for 31 patients (58.5%), with a median age of 60.0 years (IQR, 53.5–68.0 years). The largest retrospective cohort study [9], comprising 19 patients, provided aggregate data and reported a mean age of 59.8 years. Lesion laterality was reported for 19 patients (35.8%), including 12 (63.2%) with left-sided lesions, 6 (31.6%) with right-sided lesions, and 1 (5.3%) with bilateral lesions. Etiological information was available for 22 patients (41.5%). Infarction was the most common etiology, occurring in 16 patients (72.7%), followed by vertebral artery dissection in 5 (22.7%) and hemorrhage in 1 (4.5%). Preoperative tracheostomy status was available for 33 patients (62.3%), of whom 18 (54.5%) had a tracheostomy.

The interval from symptom onset to surgery was available at the patient level for 31 patients, with a median duration of 8.0 months (IQR, 4.5–15.5 months). The retrospective cohort study [9] reported a mean interval of 16.3 months for 19 patients. Preoperative swallowing status could be converted to FOIS for 34 patients, yielding a median score of 1 (IQR, 1–1). In the largest retrospective cohort study (*n* = 19) [9], the mean preoperative overall NOMS score was 1.11, indicating profound impairment of oral intake.

### 3.3. Surgical Management

The surgical management strategies are summarized in Table 3. Cricopharyngeal myotomy (CPM) was performed in all 53 patients (100%). Laryngeal suspension (LS) was the second-most frequently performed procedure, reported in 34 patients (64.2%), followed by infrahyoid muscle transection (IMT) in 8 (15.1%), pharyngeal flap (PF) in 6 (11.3%), pharyngoplasty (PP) in 6 (11.3%), and vocal fold medialization (VFM) in 5 (9.4%). Detailed information regarding the CPM technique was available for 30 patients. Bilateral CPM was the most frequently performed approach (24/30, 80.0%), followed by left-sided CPM (4/30, 13.3%) and endoscopic CPM (2/30, 6.7%). No study reported isolated right-sided CPM. Patient-level procedural data were available for 34 patients, allowing assessment of surgical combinations. CPM alone was performed in 10 patients (29.4%) [11,12,17,22,25,26,29,30,31], whereas combined procedures were performed in 24 (70.6%). The most common combination was CPM with LS plus IMT (8/34, 23.5%) [23,28], followed by CPM with LS (7/34, 20.6%) [10,13,18,19] and CPM with LS plus PP (6/34, 17.6%) [8,21]. The remaining combinations were CPM with LS plus PF, CPM with LS plus VFM, and CPM with VFM, each performed in one patient (2.9%). In the retrospective cohort of 19 patients [9], patient-level procedural combinations were unavailable; however, LS was performed in 11 patients in addition to CPM. Overall, at least 35 of the 53 patients (66.0%) underwent combined swallowing improvement procedures.

### 3.4. Postoperative Outcomes

Postoperative swallowing and airway outcomes are summarized in Table 4. Paired pre- and postoperative FOIS data were available for 27 patients. Among these patients, the median FOIS score increased from 1 (IQR, 1–1) preoperatively to 5 (IQR, 3–7) postoperatively. The proportion of patients completely dependent on tube feeding (FOIS 1) decreased from 96.3% (26/27) preoperatively to 7.4% (2/27) postoperatively. In the largest retrospective cohort study [9], only aggregate data were available, with the mean overall NOMS score improving from 1.11 preoperatively to 4.68 postoperatively. Patient-level postoperative airway outcomes were available for 12 patients. Tracheostomy closure was achieved in five patients (41.7%), whereas persistent tracheostomy was observed in four (33.3%). Three patients (25.0%) had not undergone tracheostomy before surgery.

Postoperative complications were infrequently reported across the included studies. Most studies described no major perioperative morbidity or mortality associated with swallowing improvement surgery. In the largest retrospective cohort [9], no severe procedure-related complications were reported. No cases of procedure-related mortality, wound infection, severe airway compromise, recurrent laryngeal nerve injury, or major hemorrhage were reported.

Although swallowing improvement surgery was generally associated with favorable outcomes, incomplete functional recovery was reported in a small number of patients. Inoue et al. [17] reported a patient who remained dependent on gastrostomy feeding after cricopharyngeal myotomy (CPM), whereas one of the two patients reported by Ohshima et al. [13] failed to regain functional oral intake despite surgery. Moreover, additional swallowing improvement procedures were required in some patients following insufficient functional recovery after the initial procedure. Yaguchi et al. [19] reported laryngeal suspension following unsuccessful CPM, whereas Kawamoto et al. [27] described staged laryngeal suspension with pharyngoplasty after endoscopic CPM because of persistent dysphagia.

## 4. Discussion

This scoping review provides the first comprehensive synthesis of the evidence on swallowing improvement surgery for dysphagia associated with WS. Although the available evidence consisted predominantly of case reports and small case series, several clinically important findings emerged. First, despite profound preoperative dysphagia, reflected by a median preoperative FOIS score of 1, reported postoperative swallowing outcomes suggested substantial improvement in oral intake in many patients. Second, approximately two-thirds of the reported patients underwent combined swallowing improvement procedures rather than isolated CPM, reflecting the complex and multifactorial pathophysiology of dysphagia in WS. Third, some patients required staged surgical reconstruction because the initial procedure alone did not provide sufficient functional recovery. Since CPM was performed in all reported patients, and additional procedures were often combined, it is challenging to determine the relative contribution of each procedure. The available evidence suggests that individualized, pathophysiology-directed surgical planning should be considered, rather than focusing solely on the effectiveness or superiority of any particular procedure or combination.

An important finding of this review is that most of the available evidence originated from Japan, with 66.7% of the included studies published in Japanese. Since the pioneering work of Japanese investigators more than three decades ago, extensive research has been conducted on swallowing physiology, electromyographic evaluation [32], pharyngeal manometry [33], videofluoroscopic swallowing studies (VFSS), and surgical treatment for dysphagia [34]. As a result, numerous reports describing pathophysiology-based swallowing improvement surgery have accumulated in the Japanese literature. However, because these studies were published almost exclusively in Japanese, many of these valuable clinical experiences have remained largely inaccessible to the international community. By integrating both English- and Japanese-language literature, the present review provides the first comprehensive overview of surgical management for dysphagia in WS and highlights clinical concepts that have not been widely recognized outside Japan.

In this review, men comprised 92.0% of patients with available sex data (46/50), which is a greater proportion than expected based on the general epidemiology of Wallenberg syndrome. This significant imbalance could be attributed to the small, highly selected surgical population, referral patterns, geographical concentration of the evidence, or publication bias rather than a biological sex difference in surgical need or benefit. Therefore, no sex-specific conclusions can be drawn from the available evidence.

The pathophysiology of dysphagia in WS is considerably more complex than simple UES dysfunction. The extent and location of medullary injury vary substantially among patients, resulting in marked heterogeneity of swallowing impairment [3]. The swallowing central pattern generator, located primarily within the nucleus tractus solitarius and nucleus ambiguus, coordinates the pharyngeal swallowing reflex. Injury to these structures impairs central initiation and coordination of swallowing while simultaneously affecting the nuclei of the glossopharyngeal, vagal, and hypoglossal nerves [35]. Consequently, patients with WS may present with various combinations of delayed or absent swallowing reflex, tongue weakness, velopharyngeal insufficiency, impaired pharyngeal contraction, reduced laryngeal elevation, vocal fold paralysis, impaired laryngeal sensation, and impaired UES opening [6]. The coexistence of these abnormalities explains why dysphagia in WS is highly heterogeneous and why a single surgical procedure is often insufficient.

Management should therefore begin with comprehensive assessment of the underlying swallowing pathophysiology. Reported preoperative assessments included clinical swallowing evaluation, VFSS, fiberoptic endoscopic evaluation of swallowing (FEES), and pharyngeal high-resolution manometry (HRM), although assessment methods varied among studies and were incompletely described in several older reports. More detailed physiological assessment using high-resolution manofluorography [36,37], and, when available, dynamic swallowing computed tomography (CT) with three-dimensional or virtual reality visualization [38,39] may further characterize swallowing coordination, regional pharyngeal contractility, laryngeal and hyoid movement, velopharyngeal closure, and UES opening, thereby facilitating individualized rehabilitation and surgical planning.

Reported preoperative management included swallowing rehabilitation, enteral nutritional support, airway management including tracheostomy when necessary, and, in selected cases, UES-directed treatment such as balloon dilation. Botulinum toxin injection into the cricopharyngeal muscle and endoscopic balloon dilation are minimally invasive UES-directed interventions that may improve impaired UES opening in selected patients. Botulinum toxin injection can be performed endoscopically or under direct laryngoscopy, including under general anesthesia, while balloon dilation mechanically expands the UES. Although these interventions may have important therapeutic roles, they do not involve surgical alteration of the swallowing-related anatomy and were therefore considered outside the operational definition of swallowing improvement surgery used in this review. In contrast, CPM involves surgical division of the cricopharyngeal muscle and was therefore included as a swallowing improvement surgical procedure. Postoperative rehabilitation was continued in several reports to support adaptation and advancement of oral intake. Swallowing rehabilitation remains the first-line treatment and should be individualized according to the underlying dysfunction. Depending on the specific deficits, rehabilitation may include compensatory postural strategies, swallowing exercises, tongue-strengthening exercises, effortful swallow, Mendelsohn maneuver, Shaker exercise, expiratory muscle strength training, neuromuscular electrical stimulation, and biofeedback-based therapy [37]. However, when functional recovery remains insufficient despite appropriate rehabilitation, surgical intervention should likewise be selected based on the underlying swallowing pathophysiology [40].

The present review demonstrates that surgical decision-making in WS should likewise depend on the pathophysiology rather than on the diagnosis itself. Patients with velopharyngeal insufficiency may benefit from pharyngeal flap surgery, whereas those with impaired pharyngeal contraction may require pharyngoplasty or pharyngeal reinforcement procedures. Reduced laryngeal elevation is an indication for laryngeal suspension, impaired UES opening is appropriately treated by CPM, and glottic insufficiency may require vocal fold medialization [10]. Consistent with this concept, at least 66.0% of the patients included in this review underwent combined swallowing improvement procedures rather than isolated CPM. Therefore, the diagnosis of WS itself should not determine the choice of surgical procedure. Instead, comprehensive assessment of swallowing pathophysiology should guide individualized surgical planning, with one or more swallowing improvement procedures selected and combined according to each patient’s functional impairments. Although this review focused on swallowing improvement surgery, not all patients with WS are suitable candidates for these procedures. In the series reported by Mise et al. [23], one patient with severe dysphagia underwent aspiration-prevention surgery instead of swallowing improvement surgery, suggesting that aspiration-prevention procedures should also be considered when restoration of safe swallowing is unlikely.

Another clinically important finding is the broad timing of surgical intervention. The median interval from symptom onset to surgery was eight months; however, successful outcomes were reported across a wide range of disease durations. Several patients underwent surgery within six months after onset, often because earlier restoration of oral intake or return to daily and social activities was considered desirable. Conversely, other patients achieved significant functional improvement several years after onset, suggesting that swallowing improvement surgery may remain beneficial even during the chronic phase when appropriate candidates are carefully selected. These findings indicate that surgical timing should not be determined solely by disease duration but should instead be individualized according to neurological recovery, swallowing function, rehabilitation progress, and patient preferences.

Future advances in the management of dysphagia associated with WS are expected to extend beyond surgical innovation. Novel therapeutic approaches, including central and peripheral neuromodulation and regenerative therapies targeting neural repair, may eventually restore swallowing function without the need for surgery in selected patients [41,42]. In conjunction, multimodal functional assessment combined with AI-assisted analysis has the potential to transform clinical decision-making [43]. Multimodal integration of VFSS, FEES, HRM, and dynamic swallowing CT with extended reality-based visualization may enable comprehensive characterization of patient-specific swallowing pathophysiology and support individualized treatment planning, including rehabilitation, surgical intervention, and emerging nonsurgical therapies. Such advances may ultimately shift the management of dysphagia in WS from procedure-based treatment toward precision medicine based on individualized functional assessment.

This review presents several limitations. First, the available evidence predominantly consisted of uncontrolled case reports, limited case series, and a single retrospective cohort study. Therefore, the reported improvements cannot be causally attributed to surgery and may be confounded with spontaneous neurological recovery, ongoing rehabilitation, and patient-selection effects. Second, substantial heterogeneity existed among the included studies with respect to patient characteristics, lesion severity, surgical indications, operative techniques, outcome measures, and follow-up duration, precluding quantitative meta-analysis. Third, patient-level data were unavailable for several variables due to the reporting of aggregate or incomplete data in some studies, limiting more detailed analyses. Favorable outcomes may have been preferentially reported, whereas persistent dysphagia, continued tube feeding or tracheostomy, and complications may have been underreported. Consequently, the observed postoperative distributions should not be interpreted as population-level success rates. Fourth, the evidence base was geographically concentrated, with the majority of included reports originating from Japan and numerous publications in Japanese-language domestic journals. This geographic concentration may reflect the historical development and reporting of swallowing improvement surgery in Japan, but it also limits the generalizability of the findings to other healthcare systems and populations. Finally, publication bias is inevitable, as favorable surgical outcomes are more likely to be reported than unsuccessful or complicated cases. Nevertheless, by integrating both English- and Japanese-language literature, this review provides the first comprehensive overview of swallowing improvement surgery for dysphagia associated with WS and highlights the importance of pathophysiology-based surgical decision-making. Future prospective multicenter studies integrating standardized multimodal swallowing assessment and pathophysiology-based treatment algorithms are warranted to establish evidence-based surgical strategies for dysphagia associated with WS.

## 5. Conclusions

This scoping review provides the first comprehensive synthesis of swallowing improvement surgery for dysphagia associated with Wallenberg syndrome. Reported postoperative outcomes suggest improvement in swallowing function in many carefully selected patients, although combined swallowing improvement procedures were frequently required. However, given the limited and predominantly uncontrolled evidence, the independent effect of surgery cannot be established. The available findings support individualized surgical decision-making based on the underlying swallowing pathophysiology.

## Figures and Tables

**Table 1:** Summary of Included Studies on Swallowing Improvement Surgery for Wallenberg Syndrome.

No.	Author	Year	Language	Country	Study Design	Subject No.
1	Yamane et al. [11]	1988	Japanese	Japan	Case report	1
2	Adachi et al. [12]	1996	Japanese	Japan	Case report	1
3	Ohshima et al. [13]	1998	Japanese	Japan	Retrospective case series	2
4	Inoue et al. [17]	2003	Japanese	Japan	Case report	1
5	Yamashita et al. [18]	2004	Japanese	Japan	Case series	2
6	Yaguchi et al. [19]	2006	Japanese	Japan	Case report	1
7	Kojima et al. [20]	2006	Japanese	Japan	Case report	1
8	Kiyohara et al. [21]	2007	Japanese	Japan	Case series	3
9	Kiyohara et al. [22]	2007	Japanese	Japan	Retrospective case series	2
10	Mise et al. [23]	2008	Japanese	Japan	Retrospective case series	7
11	Takahashi et al. [24]	2009	Japanese	Japan	Case report	1
12	Nair et al. [29]	2016	English	India	Case report	1
13	Verin et al. [8]	2016	English	France	Case series	2
14	Kawamoto et al. [27]	2019	Japanese	Japan	Case report	1
15	Kadosono et al. [25]	2019	Japanese	Japan	Case series	3
16	Mori et al. [26]	2020	Japanese	Japan	Case report	1
17	Shibata et al. [28]	2020	English	Japan	Retrospective case series	1
18	Brooks et al. [31]	2022	English	United States	Case report	1
19	Shenoy et al. [30]	2023	English	India	Case report	1
20	Taniguchi et al. [9]	2024	English	Japan	Retrospective cohort study	19
21	Cotaoco et al. [10]	2024	English	Japan	Case report	1

**Table 2:** Patient characteristics and preoperative clinical data.

Characteristic	Value
**Age**	
Patient-level data available, *n* (%)	31 (58.5%)
Median age, years (IQR)	60.0 (53.5–68.0)
Aggregate data only *, *n* (%) [9]	19 (35.8%)
Mean age, years *	59.8
Not reported, *n* (%) [21,22]	3 (5.7%)
**Sex**	
Patient-level data available, *n* (%)	50 (94.3%)
Male, *n* (%)	46 (92.0%)
Female, *n* (%)	4 (8.0%)
Not reported, *n* (%) [21,22]	3 (5.7%)
**Preoperative oral intake status**	
Patient-level data available, *n* (%)	34 (64.2%)
Median FOIS score (IQR)	1 (1–1)
Aggregate data only *, *n* (%) [9]	19 (35.8%)
Mean overall NOMS *	1.11
**Interval from disease onset to surgery**	
Patient-level data available, *n* (%)	31 (58.5%)
Median interval, months (IQR)	8.0 (4.5–15.5)
Aggregate data only *, *n* (%) [9]	19 (35.8%)
Mean interval, months *	16.3
Not reported, *n* (%) [18,21,28]	3 (5.7%)
**Lesion side**	
Patient-level data available ^†^, *n* (%) [10,11,12,13,17,18,19,20,21,22,24,27,29]	19 (35.8%)
Right, *n* (%)	6 (31.6%)
Left, *n* (%)	12 (63.2%)
Bilateral, *n* (%)	1 (5.3%)
Not reported, *n* (%)	34 (64.2%)
**Etiology**	
Patient-level data available ^‡^, *n* (%) [12,13,17,18,19,20,21,22,24,25,26,27,28,29,30,31]	22 (41.5%)
Infarction, *n* (%)	16 (72.7%)
Vertebral artery dissection, *n* (%)	5 (22.7%)
Hemorrhage, *n* (%)	1 (4.5%)
Not reported	31 (58.5%)
**Tracheostomy**	
Patient-level data available, *n* (%)	33 (62.3%)
Present, *n* (%)	18 (54.5%)
Not reported, *n* (%) [8,11,12,13,17,18,21,22,25,30]	20 (37.7%)

FOIS, Functional Oral Intake Scale; IQR, interquartile range; NOMS, National Outcomes Measurement System. * Aggregate data were reported only at the study level. Median values and interquartile ranges were calculated using only patient-level data. ^†^ Percentages for lesion side were calculated using the 19 patients with available lesion-side data. ^‡^ Percentages for etiology were calculated using the 22 patients with available etiological data.

**Table 3:** Surgical management.

**Procedure**	***n*** **, (%)**
Cricopharyngeal myotomy (CPM)	53 (100)
Laryngeal suspension (LS)	34 (64.2)
Infrahyoid muscle transection (IMT)	8 (15.1)
Pharyngeal flap (PF)	6 (11.3)
Pharyngoplasty (PP)	6 (11.3)
Vocal fold medialization (VFM)	5 (9.4)
**CPM technique**	***n*** **, (%)**
Patients with available procedural details	30
Bilateral [10,11,12,13,17,18,19,21,22,23,24,25,26]	24 (80.0)
Left-sided [20,25,30]	4 (13.3)
Endoscopic [27,31]	2 (6.7)
**Combinations of surgical procedures**	***n*** **, (%)**
Patients with available patient-level procedural data	34
CPM only [11,12,17,22,25,26,29,30,31]	10 (29.4)
CPM + LS + IMT [23,28]	8 (23.5)
CPM + LS [10,13,18,19]	7 (20.6)
CPM + LS + PP [8,21]	6 (17.6)
CPM + LS + PF [27]	1 (2.9)
CPM + LS + VFM [24]	1 (2.9)
CPM + VFM [20]	1 (2.9)

CPM, cricopharyngeal myotomy; IMT, infrahyoid muscle transection; LS, laryngeal suspension; PF, pharyngeal flap; PP, pharyngoplasty; VFM, vocal fold medialization. VFM includes injection laryngoplasty, type I thyroplasty, arytenoid adduction, and combinations of these procedures.

**Table 4:** Postoperative swallowing and airway outcomes.

Characteristic	Value
**Postoperative oral intake status**	
Patient-level data available, *n* (%)	27 (50.9%)
Median FOIS score (IQR)	5 (3–7)
FOIS 1, *n* (%) [13,17]	2 (7.4%)
FOIS 2, *n* (%) [18]	1 (3.7%)
FOIS 3, *n* (%) [21,22]	5 (18.5%)
FOIS 4, *n* (%)	0 (0%)
FOIS 5, *n* (%) [10,19,20,24,25,27,30,31]	10 (37.0%)
FOIS 6, *n* (%) [18]	1 (3.7%)
FOIS 7, *n* (%) [8,11,12,26,28,29]	8 (29.6%)
Aggregate data only *, *n* (%) [9]	19 (35.8%)
Mean overall NOMS	4.68
Not reported, *n* (%) [23]	7 (13.2%)
**Postoperative airway outcome**	
Patient-level data available, *n* (%)	12 (22.6%)
No tracheostomy before surgery ^†^, *n* (%) [20,25,28]	3 (25.0%)
Tracheostomy closure ^†^, *n* (%) [13,18,26,27,29]	5 (41.7%)
Persistent tracheostomy ^†^, *n* (%) [10,13,24,31]	4 (33.3%)
Not reported, *n* (%)	41 (77.4%)

FOIS, Functional Oral Intake Scale; IQR, interquartile range; NOMS, National Outcomes Measurement System. *, Aggregate data were reported only at the study level and were therefore not included in patient-level analyses. ^†^, Percentages were calculated among the 12 patients with available airway status data.

## Data Availability

No new data were created or analyzed in this study. Data sharing is not applicable to this article.

## Abbreviations

The following abbreviations are used in this manuscript:

**Abbreviation**	**Full term**
WS	Wallenberg syndrome
CPM	Cricopharyngeal myotomy
LS	Laryngeal suspension
PF	Pharyngeal flap
PP	Pharyngoplasty
IMT	Infrahyoid muscle transection
VFM	Vocal fold medialization
UES	Upper esophageal sphincter
FOIS	Functional Oral Intake Scale
NOMS	National Outcomes Measurement System
VFSS	Videofluoroscopic swallowing study
FEES	Fiberoptic endoscopic evaluation of swallowing
HRPM	High-resolution pharyngeal manometry
CT	Computed tomography
PRISMA-ScR	Preferred Reporting Items for Systematic Reviews and Meta-Analyses extension for Scoping Reviews
JBI	Joanna Briggs Institute
IQR	Interquartile range
